# Association of the central venous**-**to**-**arterial carbon dioxide difference with low cardiac output**-**related outcomes after cardiac surgery in children**:** a prospective cohort study

**DOI:** 10.3389/fped.2025.1536089

**Published:** 2025-05-23

**Authors:** Pornnicha Chaiwiriyawong, Jirayut Jarutach, Kantara Saelim, Pongsanae Duangpakdee, Polathep Vichitkunakorn, Pharsai Prasertsan

**Affiliations:** ^1^ Department of Pediatrics, Faculty of Medicine, Prince of Songkla University, Songkhla, Thailand; ^2^ Division of Cardiovascular and Thoracic Surgeon, Department of Surgery, Faculty of Medicine, Prince of Songkla University, Songkhla, Thailand; ^3^ Department of Family and Preventive Medicine, Faculty of Medicine, Prince of Songkla University, Songkhla, Thailand

**Keywords:** cardiopulmonary bypass, carbon dioxide, congenital heart disease, post-operative period, cardiac surgery, venoarterial CO_2_ difference, children

## Abstract

**Introduction:**

Low-cardiac-output syndrome (LCOS) after cardiac surgery may lead to poor postoperative outcomes. The venous-to-arterial carbon dioxide partial pressure difference (VACO_2_) showed association with poor outcomes in adults with cardiac surgery, but it's validity in pediatric population is uncertain. We evaluated the association of VACO_2_ with LCOS-related outcomes and the correlation with other surrogate markers such as lactate levels and oxygen extraction ratio.

**Methods:**

This prospective cohort study was conducted at an intensive care unit in a tertiary academic hospital. Children aged 1 day–18 years old undergoing elective cardiac surgery with cardiopulmonary bypass between August 2021 and December 2023 were included. Arterial and venous blood gases were collected at intensive care unit admission and at 6, 12, and 24 h postoperatively. The LCOS-related outcomes were defined as at least two of the following criteria being met within 24 h postoperatively: vasopressor-inotropic score ≥20, ejection fraction <50% on echocardiography, need for serious post-operative intervention, and death.

**Results:**

Of the 127 included patients (median age: 44.4 months), 37 (29.1%) had a Risk Adjustment for Congenital Heart Surgery score ≥3, and 26 (20.4%) had LCOS-related outcomes. Linear mixed model regression analysis revealed that the VACO_2_ did not significantly differ between patients with and without LCOS-related outcomes at all four time points. VACO_2_ showed a fair-to-weak correlation with the oxygen extraction ratio (*R*^2^ = 0.58; *p* < 0.001, *R*^2^ = 0.22; *p* = 0.015, and *R*^2^ = 0.19; *p* = 0.045, at 6, 12, and 24 h postoperatively, respectively) but showed no correlation with lactate levels. A persistently high VACO_2_ (≥6 mmHg) at 6 h postoperatively was significantly associated with fewer 28-day inotrope-free and intensive care unit-free days.

**Discussion:**

VACO_2_ was not significantly associated with LCOS-related outcomes in children after cardiac surgery with cardiopulmonary bypass. A persistently high VACO_2_ at 6 h postoperatively was correlated with prolonged inotrope use and a prolonged intensive care unit stay.

## 1 Introduction

Cardiopulmonary bypass (CPB) during open-heart surgery constitutes a potent stimulus that induces a systemic inflammatory response through the following mechanisms: blood contact with the CPB circuit, ischemia–reperfusion injury, heparin–protamine interaction, and surgical trauma (1). These mechanisms aggravate complement cascade activation, endotoxin release, and cytokine production, leading to low-cardiac-output (CO) syndrome (LCOS), which occurs in 5%–55% (2) of pediatric patients after cardiac surgery and frequently occurs 9–12 h postoperatively (3). Delayed recognition and treatment of LCOS can lead to multiorgan system failure and even death.

Serum lactate level, central venous oxygen saturation (ScvO_2_), and oxygen extraction ratio (O_2_ER) are commonly used to monitor LCOS after cardiac surgery, however, these parameters may be influenced by multiple factors that are unrelated to low CO. For example, hyperlactatemia can occur due to the stress response to surgery, use of beta-adrenergic agonists, hyperglycemia, or acute liver and renal failure (4); furthermore, normal or high ScvO_2_ values may be observed in the presence of mitochondrial dysfunction or the peripheral shunting effect (5). Some studies in sepsis population demonstrated unclear benefits of lactate levels and ScvO_2_ for guiding therapy (6, 7). Recently, a novel bedside biomarker, venous-to-arterial carbon dioxide (CO2) partial pressure difference (VACO2), has emerged, suggesting a potential utility as an adjunctive marker to facilitate the guidance of therapeutic interventions in sepsis (5, 8).

The VACO_2_ theoretically measures the circulatory clearance of tissue CO_2_ and is inversely correlated with CO (9). VACO_2_ is a better surrogate indicator of stagnant dysoxia than of dysoxia caused by hypoxia, anemia, or cytopathic pathology (9). A high VACO_2_ at the time of sepsis diagnosis in adult patients exhibited a significant correlation with both the cardiac index and other tissue perfusion parameters such as lactate levels and ScvO_2_ (10). A recent meta-analysis of 21 studies with 2,155 critically ill adult patients with both surgical and medical conditions demonstrated an association between high VACO_2_ and CO and linked this to mortality (11). However, only four of these 21 studies focused on cardiac surgery, and they all reported different associations with unfavorable outcomes. Chen et al. (12) conducted propensity-matched analysis in 228 individuals post cardiac surgery with CPB and reported a significant influence of high VACO_2_ on adverse outcomes with superior discrimination power than arterial lactate. Furthermore, two prospective adult studies (13, 14) indicated that high VACO_2_ is an independent predictor of major postoperative complication after multivariate analyses. Owing to the limited data on VACO_2_ in pediatric patients after cardiac surgery with CPB, the relevance of VACO_2_ as a predictor of poor outcomes in this population remains unclear.

In this study, we aimed to evaluate the association between VACO_2_ and LCOS-related outcomes in children undergoing cardiac surgery with CPB and determine its correlation with other bedside surrogate markers. The secondary objective was to examine the association between VACO_2_ and postoperative outcomes.

## 2 Materials and methods

### 2.1 Study design, setting, and participants

This prospective cohort study was performed in an eight-bed pediatric intensive care unit (PICU) at Songklanagarind Hospital, Hat Yai, Songkhla. Children aged 1 day–18 years with congenital or acquired cardiac disease who underwent elective open cardiac surgery with CPB and were admitted to the PICU postoperatively were included in the study. The exclusion criteria were as follows: preterm infants (gestational age <37 weeks), weight <2 kg, inability to wean off CPB, requirement for extracorporeal membrane oxygenation (ECMO) before leaving the operating room, and absence of arterial and central lines postoperatively. This study was approved by the Institutional Review Board of the Faculty of Medicine, Prince of Songkla University (Institutional Review Board approval number 64-299-1-1; date of approval: August 3, 2021). The study was conducted in accordance with Good Clinical Practice and the Helsinki Declaration of 1975.

### 2.2 Surgical procedures

Anesthesia was initiated according to our standardized protocol. Following incision, heparin was administered intravenously at a dosage of 3 mg/kg to achieve an activated clotting time (ACT) exceeding 400 s. ACT was monitored at 30-min intervals throughout the procedure, with an additional heparin dose of 3 mg/kg provided if the ACT fell below 400 s. Intraoperative corticosteroids were administered intravenously, based on individual anesthesiologist preferences. The priming solution for the CPB circuit included either Ringer's lactate or normal saline with mannitol, along with 20% albumin for patients weighing less than 10 kg. Leukocyte-depleted packed red blood cells were utilized when the preoperative hematocrit was below 30%. CPB was performed using a CAPIOX® FX05 Oxygenator and Stockert S5 pump, following the *α*-stat strategy at a target temperature of 28°C. Hypothermia was induced to varying degrees; depending on the surgical procedure. Antegrade cold blood cardioplegia was administered at a volume of 20 ml/kg to achieve cardiac arrest, with an additional 10 ml/kg given if aortic cross-clamp time exceeded 20 min. Pump flow rates were maintained between 100 and 150 ml/kg/min for infants and 2.5–3.0 L/m^2^/min for older patients: adjusted according to age-appropriate mean arterial pressure. Arteriovenous modified ultrafiltration was selectively applied, based on the consensus of the surgical team and perfusionist, and performed for 5 min after separating from CPB. At the conclusion of CPB intravenous protamine sulfate was administered to reverse heparin anticoagulation.

### 2.3 Data collection and measurement

After receiving written informed consent from the patients’ parents or legal guardians before operation, 0.3 ml blood samples were simultaneously collected from both the arterial and internal jugular central lines. This sampling was repeated at four different time points after patient arrival in the PICU: at PICU arrival (T0), 6-h (T6), 12-h (T12), and 24-h (T24) post operation. Arterial and venous blood gas readings were accepted if the samples were collected within 5 min of each other. A 1 h time gap in the collection of blood samples from the research schedule was allowed owing to unpredictable intensive care unit occupancy. Blood samples were analyzed within 1 min of blood collection using an arterial blood gas analysis machine (ABL 800 Basic Radiometer; Copenhagen, Denmark) located within the PICU. The attending staff independently provided standard post-cardiac surgery care, including fluid resuscitation, vasopressor and inotropic medication, and steroid administration without the researcher's involvement. If either the central or arterial lines were displaced within 24 h postoperatively, the remaining data were recorded as missing.

Data, including general baseline characteristics, intraoperative parameters, postoperative interventions, and outcomes, were collected until the patient was discharged. The Risk Adjustment for Congenital Heart Surgery tool (15) was used to classify the risk of mortality after congenital cardiac surgery. The vasoactive-inotropic score (VIS) was calculated using the formula by Gaies et al. (16) as follows:

$$\begin{array}{rl} \mathrm{VIS}= & \mathrm{Dopamine}\;\mathrm{dose}\;(\mu g/\mathrm{kg}/\min)\\ & +\mathrm{dobutamine}\;\mathrm{dose}\;(\mu g/\mathrm{kg}/\min)+100\\ & \times \mathrm{epinephrine}\;\mathrm{dose}\;(\mu g/\mathrm{kg}/\min)\;+\;10\\ & \times \mathrm{milrinone}\;\mathrm{dose}\;(\mu g/\mathrm{kg}/\min)\;+\;100\\ & \times \mathrm{norepinephrine}\;\mathrm{dose}\;(\mu g/\mathrm{kg}/\min)\;+10000\\ & \times \mathrm{vasopressin}\;\mathrm{dose}\;(U/\mathrm{kg}/\min) \end{array}$$

Acute kidney injury was diagnosed according to the Kidney Disease-Improving Global Outcomes guidelines, 2012 (17), based on the presence of any of the following: a >0.3 g/dl increase in the serum creatinine level within 48 h, >1.5 times increase in the serum creatinine level from a known baseline value or one recorded within the preceding 7 days, and urinary volume <0.5 ml/kg/h for 6 h. The 28-day ventilator-free days (VFDs), 28-day inotrope-free days, and 28-day ICU-free days (IFDs) were defined as the number of days that the patient survived without invasive ventilation, inotropic drugs, or ICU admission during the first 28 days postoperatively, with the day after the first postoperative night considered as day 1. These variables were counted as zero for non-survivors.

The independent variables studied included the bedside surrogate markers (VACO_2_, O_2_ER, and lactate level) that were measured postoperatively. ScvO_2_ was not evaluated because the participants’ single-ventricle physiology might have interfered with ScvO_2_ interpretation. VACO_2_ was calculated as the central venous minus arterial CO_2_ level and O_2_ER as the ratio between the difference in arterial and central venous oxygen saturation divided by the arterial oxygen saturation.

The primary outcomes were LCOS-related outcomes, which was consisted of at least two of the following criteria within 24 h postoperatively: (1) VIS ≥20; (2) left ventricular ejection fraction <50% on echocardiography; (3) any unplanned surgery or intervention, cardiac arrest, or utilization of ECMO; and (4) death. The secondary outcomes included: 28-day VFDs, 28-day inotrope-free days, 28-day IFDs, percentage of morbidities (reintubation, significant arrhythmic events requiring medication or intervention, acute kidney injury, renal replacement therapy, and neurological complications), and mortality rate.

### 2.4 Statistical analyses

Statistical analyses involved descriptive analysis of means (standard deviations) for normally distributed continuous data, medians (interquartile ranges) for non-normally distributed continuous data, and percentages for categorical data. The Student's *t*-test or Mann–Whitney *U* test was used for intergroup comparison of continuous data, depending on the pattern of data distribution. Categorical data were compared using the chi-squared or Fisher's exact test, as indicated. Correlation analysis between bedside parameters was performed using Pearson's and Spearman's correlation coefficients for parametric and non-parametric variables, respectively. Linear mixed-model regression was used to compare the postoperative laboratory (lactate, VACO_2_, and O_2_ER) values between the LCOS and no-LCOS groups over time with adjusted confounder variables that might affect outcome (age, RACHS, type of repair, CPB time, intraoperative corticosteroid, intraoperative fluid balance). Receiver operating characteristic curves were used to evaluate performance of VACO_2_ on discriminating LCOS-related outcomes. Subgroup analysis was performed according to patient's age, type of repair, and RACHS score. Statistical significance was set at *p* < 0.05. All analyses were conducted using R version 4.3.1 (The R Foundation for Statistical Computing, Vienna, Austria).

### 2.5 Ethics statement

Ethical approval for this study was obtained from the Ethics Committee of the Faculty of Medicine, Prince of Songkla University, Songkhla, Thailand.

## 3 Results

### 3.1 Baseline characteristics of participants

Between August 2021 and December 2023, 136 patients underwent open-heart surgery. Nine patients were excluded from the study, which included five patients who could not be weaned off CPB postoperatively and four patients who underwent emergency operations. The final analysis included 127 patients, of which 26 (20.4%) developed LCOS-related outcomes within 24 h postoperatively. Five patients (3.9%) required re-operation or reintervention, seven (5.5%) required ECMO, three (2.4%) developed cardiac arrest, and seven (5.5%) died. Factors significantly associated with LCOS-related outcomes included preoperative sepsis, a high Risk Adjustment for Congenital Heart Surgery score, long CPB time, long aortic clamp time, high amount of blood transfusion, and intraoperative corticosteroid administration (Table 1). The rates of postoperative intervention, including systemic steroid administration (60.4% vs. 24.3%, *p* < 0.01), blood transfusion [19.6 [10.0–34.6] vs. 6.4 [0–13.5] ml/kg, *p* < 0.01], and renal replacement therapy (13% vs. 0%, *p* = 0.002), as well as those of end-organ dysfunction and death, were higher in the LCOS group than in the no-LCOS group (Supplementary Table 1).

**Table 1:** Comparison of baseline characteristics and intraoperative parameters between patients with and without LCOS**-**related outcomes (*n* **=** 127).

Characteristic	LCOS-related outcomes (*n* = 26)	No LCOS-related outcomes (*n* = 101)	*p*-value
Age (months), median (IQR)	5.4 (0.8–74.7)	32.5 (11.9–69.7)	0.053
Genetic abnormalities, *n* (%)	2 (7.7)	16 (15.8)	0.363
Preoperative sepsis, *n* (%)	12 (46.2)	16 (15.8)	0.002
Previous cardiac surgery, *n* (%)	7 (26.9)	32 (31.7)	0.817
Single-ventricle repair, *n* (%)	5 (19.2)	18 (17.8)	1.000
RACHS score, *n* (%)			<0.001
<3	8 (30.8)	82 (82.2)	
≥3	18 (69.2)	19 (17.8)	
Cardiopulmonary bypass time (min), median (IQR)	152.0 (136.8, 244.2)	84.0 (52.0, 140.0)	<0.001
Aortic clamp time (min), median (IQR)	111.5 (77.2, 164.2)	47.0 (22.0, 82.8)	<0.001
Intraoperative blood transfusion (ml/kg), median (IQR)	60.4 (43.5, 91.9)	37.0 (23.6, 54.7)	<0.001
Intraoperative fluid balance (ml/kg), median (IQR)	21 (2.3, 38.4)	18.2 (8.0, 37.7)	0.998
Intraoperative steroid use, *n* (%)	6 (23.1)	6 (5.9)	0.016

IQR, interquartile range; LCOS, low-cardiac-output syndrome; RACHS, Risk Adjustment for Congenital Heart Surgery.

### 3.2 Association between VACO_2_ and outcomes after cardiac surgery

**Figure 1:**
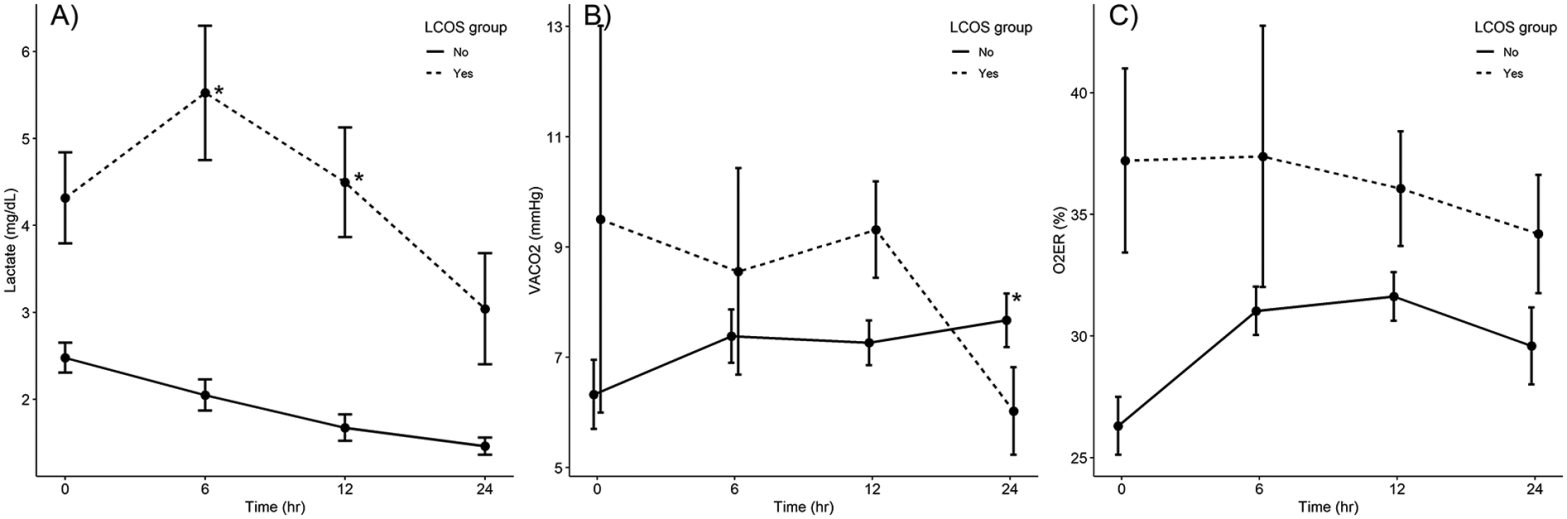
Comparison of laboratory values **(A)** lactate level, **(B)** VACO_2_, **(C)** O_2_ER between patients with and without LCOS-related outcomes at four time points (*N* = 127). *p < 0.05, in the linear mixed-model regression analysis. AVO_2_, arteriovenous oxygen saturation difference LCOS, low-cardiac-output syndrome; VACO_2_, venous-to-arterial carbon dioxide partial pressure difference.

The patterns of laboratory values at the four time points are illustrated in Figure 1. The overall values of three parameters in the LCOS group were higher than those in the non-LCOS group at all four time points, with the exception for the VACO_2_ at 24 h post operation. Univariate analysis performed to compare bedside surrogate marker values between patients with and without poor LCOS-related outcomes revealed that the LCOS group had significantly higher VACO_2_ values at 12 h postoperatively, higher lactate levels at all four time points, and higher O_2_ER values at PICU admission and at 6 h postoperatively than the no-LCOS group. Analysis of the overall unadjusted area under the curve (AUC) of VACO_2_ was 0.58–0.64 which was inferior to serum lactate levels in predicting LCOS events (Table 2).

**Table 2:** Comparison of laboratory values between patients with and without LCOS**-**related outcomes (*n* **=** 127).

Characteristic	LCOS-related outcomes (*n* = 26)	No LCOS-related outcomes (*n* = 101)	*p*-value	AUC (95% CI)
VACO_2_ (mmHg)				
T0	8.3 (3.2, 14.1)	6.8 (2.8, 10.2)	0.240	0.58 (0.43–0.72)
T6	9.4 (5.9, 12.0)	7.8 (5.3, 9.4)	0.175	0.59 (0.44–0.74)
T12	9.8 (6.4, 11.8)	7.2 (5.0, 9.8)	0.027	0.64 (0.52–0.77)
T24	5.8 (3.1, 11.8)	7.5 (5.8, 9.7)	0.078	0.62 (0.48–0.77)
Lactate level (mmol/L)				
T0	3.2 (2.2, 5.8)	1.9 (1.4, 2.2)	<0.001	0.74 (0.63–0.84)
T6	4.8 (2.5, 8.2)	1.4 (1.1, 2.2)	<0.001	0.84 (0.75–0.93)
T12	3.8 (2.4, 5.7)	1.2 (0.8, 1.9)	<0.001	0.83 (0.73–0.93)
T24	1.8 (1.3, 2.9)	1.3 (0.9, 1.7)	0.003	0.71 (0.58–0.83)
O_2_ER (%)				
T0	33.7 (24.7, 52.0)	24.6 (19.7, 31.9)	0.006	0.68 (0.54–0.81)
T6	35.3 (28.5, 55.7)	30.4 (25.3, 37.6)	0.017	0.66 (0.52–0.80)
T12	36.1 (11.8)	31.6 (10.0)	0.058	0.61 (0.47–0.74)
T24	31.8 (28.3, 40.0)	30.3 (24.1, 35.7)	0.210	0.59 (0.45–0.73)

Data are presented as means with standard deviations or medians with interquartile ranges. AUC, area under the curve; CI, confidence interval; LCOS, low-cardiac-output syndrome; O_2_ER, oxygen extraction ratio; ScvO2, central venous oxygen saturation; VACO_2_, venous-arterial carbon dioxide partial pressure difference.

After performing linear mixed-model regression analysis, the relationship between lactate change and the LCOS occurrence was significantly at 6 h and 12 h postoperatively (*p* < 0.05), whereas the VACO_2_ change was significant at 24 h postoperatively (*p* = 0.02) and there was no relationship between O_2_ER change at all four time points (Figure 1).

To achieve the secondary study objective, we used a 6-mmHg cut-off, as described previously (9), to categorize the 6-h postoperative VACO_2_ values into two groups. The results revealed significantly fewer 28-day inotrope-free days and 28-day IFDs in patients with a VACO_2_ ≥6 mmHg than in patients with a VACO_2_ <6 mmHg (24.0 [22.0–27.0] vs. 26.5 [23.0–28.0] days, *p* = 0.03 and 23.0 [18.2–26.0] vs. 24.0 [22.0–27.0] days, *p* = 0.03, respectively) (Table 3). Subgroup analysis in patients with biventricular physiology and with aged more than one-month old revealed insignificant different outcomes (Supplementary Tables 2, 3).

**Table 3:** Comparison of outcomes between patients with a VACO_2_ <6 mmHg and ≥6 mmHg at 6 h after PICU admission (*n* **=** 124).

Characteristic	VACO_2_ <6 mmHg (*n* = 38)	VACO_2_ ≥6 mmHg (*n* = 86)	*p*-value
LCOS-related poor outcomes, *n* (%)	7 (18.4)	18 (20.9)	0.938
VIS >20	18 (47.4)	46 (53.5)	0.664
Reintervention	3 (7.9)	2 (2.3)	0.167
ECMO	1 (2.6)	5 (5.8)	0.666
Cardiopulmonary arrest	0 (0)	3 (3.5)	0.552
Reintubation, *n* (%)	3 (7.9)	9 (10.5)	0.754
28-day ventilation-free days, median (IQR)	27.0 (25.2–28.0)	27.0 (24.0–28.0)	0.085
28-day inotrope-free days, median (IQR)	26.5 (23.0–28.0)	24.0 (22.0–27.0)	0.027
28-day ICU-free days, median (IQR)	24.0 (22.0–27.0)	23.0 (18.2–26.0)	0.031
Significant arrhythmias, *n* (%)	7 (18.4)	18 (20.9)	0.938
Acute kidney injury, *n* (%)	2 (5.3)	16 (18.8)	0.091
Renal replacement therapy, *n* (%)	1 (2.6)	6 (7.0)	0.437
Neurological complication, *n* (%)	1 (2.6)	4 (4.7)	1.000
Death, *n* (%)	1 (2.6)	6 (7.0)	0.437

ECMO, extracorporeal membrane oxygenation; ICU, intensive care unit; IQR, interquartile range; LCOS, low cardiac output syndrome; PICU, pediatric intensive care unit; VACO_2,_ venous-to-arterial carbon dioxide partial pressure difference; VIS, vasoactive-inotropic score.

*N* = 124 because three patients required ECMO or died at 6 h after PICU admission.

### 3.3 Correlations between VACO_2_ and other parameters

**Figure 2:**
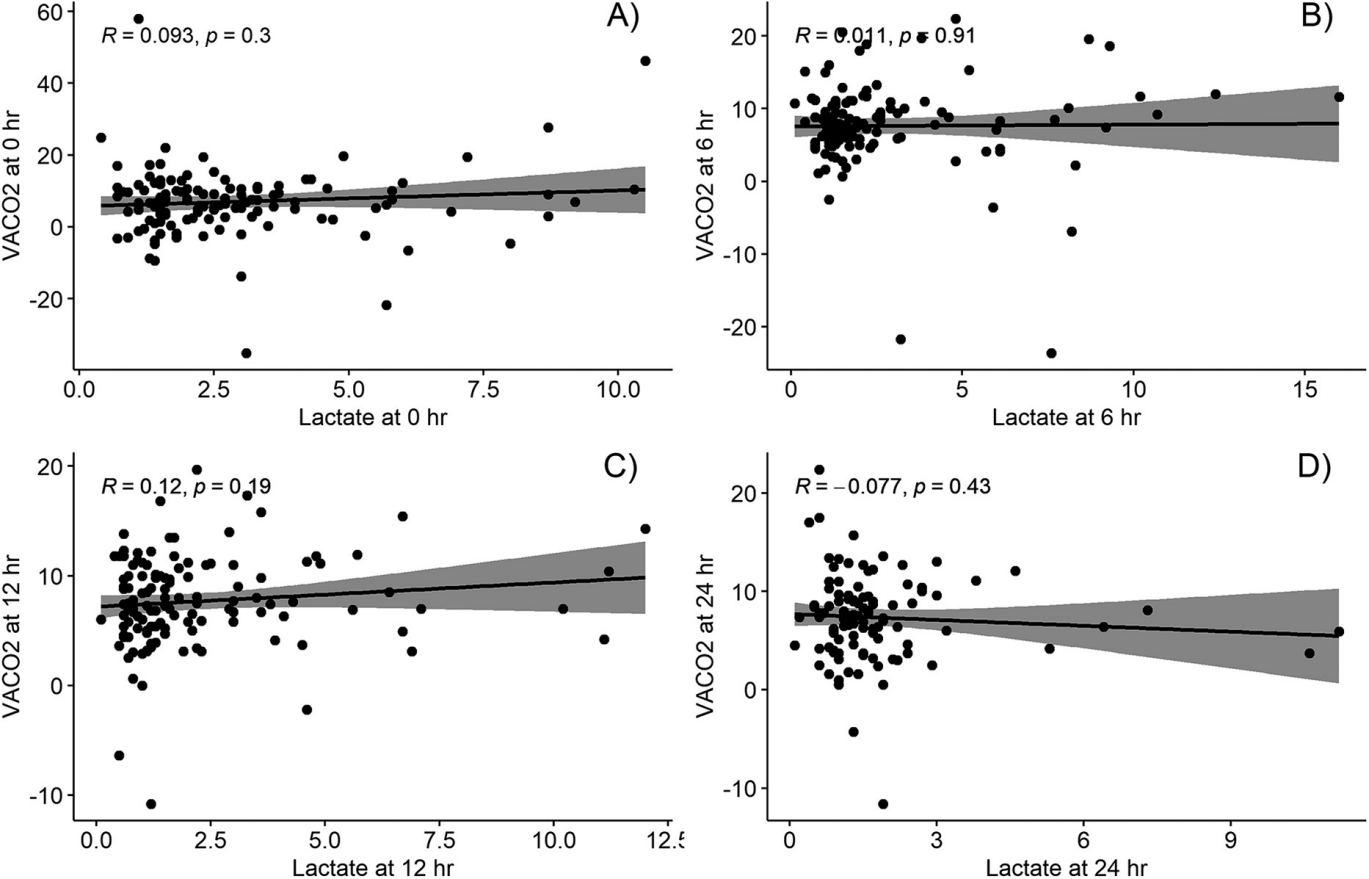
Correlation between venous-to-arterial carbon dioxide partial pressure difference and lactate at different timepoints; **(A)** at intensive care arrival, **(B)** at 6 h post operation, **(C)** at 12 h post operation, and **(D)** at 24 h post operation. VACO_2_, venous-to-arterial carbon dioxide partial pressure difference.

**Figure 3:**
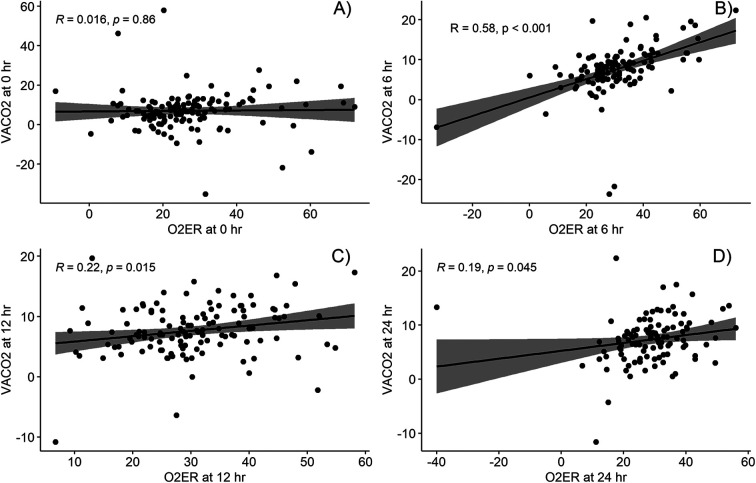
Correlation between venous-to-arterial carbon dioxide partial pressure difference and oxygen extraction ratio at different timepoints; **(A)** at intensive care arrival, **(B)** at 6 h post operation, **(C)** at 12 h post operation, and **(D)** at 24 h post operation. O_2_ER, oxygen extraction ratio; VACO_2,_ venous-to-arterial carbon dioxide partial pressure difference.

The correlations between VACO_2_ and the other parameters (lactate level and O_2_ER) at the four time points are shown in Figures 2, 3. While VACO_2_ did not show a significant correlation with lactate levels, it demonstrated a moderate correlation with O_2_ER at 6 h postoperatively (*r* = 0.58; *p* < 0.001) and a weak correlation with O_2_ER at 12 and 24 h postoperatively (*r* = 0.22; *p* = 0.015 and *r* = 0.19; *p* = 0.045, respectively).

## 4 Discussion

This study explores the predictive value of VACO_2_ in children undergoing cardiac surgery with CPB at four time points (PICU admission and 6, 12, and 24 h postoperatively) and compares it with that of other bedside indicators. The primary finding is that VACO_2_ shows no association with LCOS-related outcomes and has lower predictive capability than arterial lactate levels. Specifically, an elevated VACO_2_ at 6 h postoperatively correlates with fewer 28-day inotrope-free days and IFDs.

Our negative findings were similar to Akamatsu et al. (18) regarding the correlation with other surrogates and the association between a VACO_2_ ≥6 mmHg and unfavorable outcomes (prolonged extubation, duration of hospitalization, and mortality). That study was conducted retrospectively in 114 pediatric patients aged up to 18 years with single blood sampling of the VACO_2_ at the time of admission. However, this is in contrast with the findings of Rhodes et al. (19), who found a high VACO_2_ at PICU admission was linked to LCOS-related outcomes (high inotropic score, cardiac arrest, ECMO use, and unplanned surgical interventions within 48 h of PICU admission). They also found VACO_2_ to have a predictive ability equal to that of lactate levels and superior to that observed in the present study (AUC for VACO_2_, 0.69 and AUC for lactate levels; 0.64). Another prospective study, conducted in 69 China infants, linked VACO_2_ >12.3 mmHg within 42 h postoperatively to prolonged ventilator use and ICU stay (20). These contrast results could be attributed to different age groups within the targeted population, as these two studies were conducted in infants, whereas our study and that of Akamatsu et al. (18) were carried out in children aged up to 18 years. Studies in adults also reflect variability in the predictive power of VACO_2_. No association with outcomes was noted in some studies (21–23), while significant links to mortality were reported in others (12, 14, 24, 25). Overall, the discriminatory performance of VACO_2_ in adults ranges widely, from 0.52 to 0.83; this is similar to our findings.

The lack of significant association between VACO_2_ and LCOS-related outcomes in this study may be attributed to several factors. First, circulatory dynamics differ between children and adult's cardiac condition for operation. This study included all pediatric patients across a wide range of ages and congenital cardiac anomalies, encompassing both uni- and biventricular systems. Although subgroup analysis was performed in biventricular physiology group, residual lesion did not take into account. Residual cardiac lesions following total correction or staged reconstruction surgeries in children could influence VACO_2_ while most cardiac surgery in adults are performed within the biventricular system which do not interfere with mixing cardiac lesions. This was supported by a study on pediatric participants with sepsis who had biventricular systems demonstrated a significant association between VACO_2_ ≥6 mmHg and mortality (10).

Second, VACO_2_ measurements may be influenced by conditions such as hyperventilation and hyperoxia in the post-cardiac surgery setting. This might transiently widen the CO_2_ gap through acute decreases in arterial CO_2_ levels and increased venous CO_2_ levels (26, 27), potentially limiting the utility of VACO_2_ as a reliable marker for predicting LCOS-related outcomes.

Third, the clinical outcomes following pediatric cardiac surgery are multifaceted and not solely attributable to low CO. Factors such as anemia due to intraoperative blood loss or mitochondrial dysfunction due to CPB effects may not necessarily result in an abnormal VACO_2_, although they can significantly impact poor outcomes (9). Fourth, the timing of the VACO_2_ evaluation in previous studies differed from that in this study. Most of the adult and pediatric studies in cardiac surgery assessed VACO_2_ values at the time of admission. In this study, we hypothesized that the evaluation time point at the 6-h postoperative is appropriate for identifying the consequences of LCOS after patients received initial stabilization without the effect of anesthetic in the operating room. Moreover, the inflammatory response that leads to LCOS reaches peak effect at 9–12 h postoperatively (2). Because of the rapid responsiveness of VACO_2_ to a low circulatory flow state, persistently high VACO_2_ in a specific period might be better indicate outcomes than single values at admission time.

Our findings for the correlation between VACO_2_ and other parameters are similar to those of Rhodes et al. and Singh et al. (19, 28); we observed a moderate correlation of VACO_2_ with O_2_ER but no significant correlation with lactate levels. Similarly, the results of two studies of post cardiac surgery in adults concur with the findings in that VACO_2_ had weak or no correlation with lactate levels (21, 29), despite there being a strong correlation with CO (12, 20). Although Castanuela et al. (30) reported a moderate correlation of VACO_2_ with lactate levels at 12 h postoperatively (*R*^2^ = 0.59, *p* < 0.001), the correlation became weaker when analyzing the total number of sample collected (*R*^2^ = 0.25, *p* < 0.001). The moderate correlation of VACO_2_ with O_2_ER but not with lactate levels could be because O_2_ER and VACO_2_ change faster than lactate levels in response to circulatory flow changes.

To our knowledge, this is the first prospective study involving rigorous VACO_2_, O_2_ER, and lactate level assessment at multiple time points following open-heart surgery in a pediatric population. Although this study involved only 127 pediatric patients, the sample size was larger than previous studies. However, it has some limitations. First, the study encompassed a wide variety of patients in terms of age, cardiac abnormalities, and surgical procedures, potentially diluting the significance of the results. Subgroup analysis was performed and remained insignificant different outcomes. The power could be enhanced by increasing the sample size to perform subgroup analyses. Second, the applicability of the gold standard method such as thermodilution method via Swan–Ganz catheterization or transesophageal echocardiography for diagnosing LCOS is limited to children, especially in postoperative states. There has been no consensus on the diagnostic criteria for LCOS in the pediatric population yet. The composite criteria we had set would identify the closest consequences from LCOS. Therefore, we are unable to endorse utilizing the VACO_2_ as the bedside parameter in children who have undergone cardiac surgery with CPB, as there has not been strong evidence from the prospective study.

## 5 Conclusions

High level of VACO_2_ was not significantly associated with LCOS-related outcomes in children who underwent cardiac surgery with CPB. However, a persistently high VACO_2_ at 6 h postoperatively was related to prolonged inotropic use and a prolonged ICU stay. Further research on VACO2 as an adjunctive diagnostic parameter by combining with lactate level might improve diagnostic accuracy.

## Data Availability

The raw data supporting the conclusions of this article will be made available by the authors, without undue reservation.
