# Supplementary material for: Association of the central venous-to-arterial carbon dioxide difference with low cardiac output-related outcomes after cardiac surgery in children: a prospective cohort study
Source: Front Pediatr. 2025 May 23;13:1536089. doi: 10.3389/fped.2025.1536089 (PMC12141210; doi:10.3389/fped.2025.1536089)
Supplement: Supplementary file 1 [file Table1.docx]

Supplementary Material

# Supplementary Figures and Tables

## Supplementary Table

**Supplementary Table 1. Comparison of postoperative interventions and outcomes between patients with and without LCOS-related outcomes (n=127)**

| Characteristics | LCOS-related outcomes  (n=26) | No LCOS-related outcomes  (n=101) | *p*-value |
| --- | --- | --- | --- |
| Postoperative steroid use, n (%) | 32 (60.4) | 18 (24.3) | <0.001 |
| Postoperative blood transfusion, (mL/kg), median (IQR) | 19.6 (10.0–34.6) | 6.4 (0, 13.5) | <0.001 |
| Inhaled nitric oxide usage, n (%) | 8 (15.1) | 5 (6.8) | 0.218 |
| Reintubation, n (%) | 5 (9.4) | 8 (10.8) | 1.000 |
| 28-day ventilator-free days, median (IQR) | 24.0 (14.0–27.0) | 27.5 (27.0–28.0) | <0.001 |
| 28-day inotrope-free days, median (IQR) | 22.0 (10.0–24.0) | 27.0 (25.0–28.0) | <0.001 |
| 28-day ICU-free days, median (IQR) | 21.0 (9.0–23.0) | 25.5 (22.5–27.0) | <0.001 |
| Significant arrhythmia, n (%) | 17 (32.1) | 9 (12.2) | 0.012 |
| Acute kidney injury, n (%) | 14 (26.4) | 4 (5.5) | 0.002 |
| Renal replacement therapy, n (%) | 7 (13.2) | 0 (0) | 0.002 |
| Neurological complication, n (%) | 5 (9.4) | 0 (0) | 0.011 |
| Death, n (%) | 7 (13.2) | 0 (0) | 0.002 |

ICU, intensive care unit; IQR, interquartile range; LCOS, low-cardiac-output syndrome

**Supplementary Table 2. Comparison of outcomes between patients with a VACO_2_ <6 mmHg and ≥6 mmHg at 6 h after PICU admission in patients aged more than one month old (n=113)**

| Characteristic | VACO_2_ <6 mmHg  (n=36) | VACO_2_ ≥6 mmHg  (n=77) | *p*-value |
| --- | --- | --- | --- |
| LCOS-related poor outcomes, n (%) | 6 (16.7) | 12 (15.6) | 1.000 |
| VIS >20 | 16 (44.4) | 40 (51.9) | 0.558 |
| Reintervention, n (%) | 3 (8.3) | 2 (2.6) | 0.325 |
| ECMO, n (%) | 0 (0) | 3 (3.9) | 0.550 |
| Cardiopulmonary arrest | 0 (0) | 2 (2.6) | 1.000 |
| Reintubation, n (%) | 3 (8.3) | 9 (11.7) | 0.749 |
| 28-day ventilation-free days, median (IQR) | 27.5 (26.0–28.0) | 27.0 (24.0–28.0) | 0.108 |
| 28-day inotrope-free days, median (IQR) | 27.0 (23.0–28.0) | 25.0 (22.0–27.0) | 0.019 |
| 28-day ICU-free days, median (IQR) | 24.5 (22.0–27.0) | 23.0 (19.0–26.0) | 0.021 |
| Significant arrhythmias, n (%) | 5 (13.9) | 17 (22.1) | 0.442 |
| Acute kidney injury, n (%) | 1 (2.8) | 13 (17.1) | 0.035 |
| Renal replacement therapy, n (%) | 0 (0) | 4 (5.2) | 0.305 |
| Neurological complication, n (%) | 0 (0) | 3 (3.9) | 0.550 |
| Death, n (%) | 1 (2.8) | 4 (5.2) | 1.000 |

ECMO, extracorporeal membrane oxygenation; ICU, intensive care unit; IQR, interquartile range; LCOS, low cardiac output syndrome; PICU, pediatric intensive care unit; VACO_2,_ venous-to-arterial carbon dioxide partial pressure difference; VIS, vasoactive-inotropic score

**Supplementary Table 3. Comparison of outcomes between patients with a VACO_2_ <6 mmHg and ≥6 mmHg at 6 h after PICU admission in patients with biventricular physiology (n=101)**

| Characteristic | VACO_2_ <6 mmHg  (n=31) | VACO_2_ ≥6 mmHg  (n=70) | *p*-value |
| --- | --- | --- | --- |
| LCOS-related poor outcomes, n (%) | 5 (16.1) | 15 (21.4) | 0.730 |
| VIS >20 | 14 (45.2) | 32 (45.7) | 1.000 |
| Reintervention, n (%) | 1 (3.2) | 2 (2.9) | 1.000 |
| ECMO, n (%) | 1 (3.2) | 3 (4.3) | 1.000 |
| Cardiopulmonary arrest | 0 (0) | 2 (2.9) | 1.000 |
| Reintubation, n (%) | 3 (9.7) | 9 (12.9) | 0.751 |
| 28-day ventilation-free days, median (IQR) | 27.0 (25.0–28.0) | 27.0 (24.0–28.0) | 0.113 |
| 28-day inotrope-free days, median (IQR) | 27.0 (23.0–28.0) | 24.0 (22.0–27.0) | 0.116 |
| 28-day ICU-free days, median (IQR) | 24.0 (22.0–27.0) | 23.0 (19.0–26.0) | 0.153 |
| Significant arrhythmias, n (%) | 6 (19.4) | 16 (22.9) | 0.895 |
| Acute kidney injury, n (%) | 2 (6.5) | 10 (14.5) | 0.333 |
| Renal replacement therapy, n (%) | 1 (3.2) | 4 (5.7) | 1.000 |
| Neurological complication, n (%) | 1 (3.2) | 2 (2.9) | 1.000 |
| Death, n (%) | 1 (3.2) | 4 (5.7) | 1.000 |

ECMO, extracorporeal membrane oxygenation; ICU, intensive care unit; IQR, interquartile range; LCOS, low cardiac output syndrome; PICU, pediatric intensive care unit; VACO_2,_ venous-to-arterial carbon dioxide partial pressure difference; VIS, vasoactive-inotropic score
